# Native T1 is predictive of cardiovascular death/heart failure events and all-cause mortality irrespective of the patient’s volume status

**DOI:** 10.3389/fcvm.2023.1091334

**Published:** 2023-02-14

**Authors:** Julia Treiber, Carla S. Hausmann, Jan Sebastian Wolter, Ulrich Fischer-Rasokat, Steffen D. Kriechbaum, Christian W. Hamm, Eike Nagel, Valentina O. Puntmann, Andreas Rolf

**Affiliations:** ^1^Department of Cardiology, Kerckhoff Heart and Thorax Center, Bad Nauheim, Germany; ^2^German Center for Cardiovascular Research (DZHK), Partner Site Rhine-Main, Bad Nauheim, Germany; ^3^Justus Liebig University of Giessen, Giessen, Germany; ^4^Institute for Experimental and Translational Cardiovascular Imaging, University Hospital Frankfurt am Main, Frankfurt, Germany

**Keywords:** cardiovascular magnetic resonance (CMR), T1, plasma volume, prognosis, heart failure, T1-mapping

## Abstract

**Background:**

Native T1 has become a pivotal parameter of tissue composition that is assessed by cardiac magnetic resonance (CMR). It characterizes diseased myocardium and can be used for prognosis estimation. Recent publications have shown that native T1 is influenced by short-term fluctuations of volume status due to hydration or hemodialysis.

**Methods:**

Patients from a prospective BioCVI all-comers clinical CMR registry were included, and native T1 and plasma volume status (PVS) were determined according to Hakim’s formula as surrogate markers of patient volume status. The primary endpoint was defined as combined endpoint of cardiovascular death or hospitalization for heart failure events, the secondary endpoint was defined as all-cause mortality.

**Results:**

A total of 2,047 patients were included since April 2017 [median (IQR); age 63 (52–72) years, 33% female]. There was a significant although weak influence of PVS on native T1 (*β* = 0.11, *p* < 0.0001). Patients with volume expansion (PVS > −13%) showed significantly higher values for tissue markers than non-volume-overloaded patients [PVS ≤ −13%; median (IQR); native T1 1,130 (1,095–1,170) vs. 1,123 (1,086–1,166) ms, *p* < 0.003; and T2 39 (37–40) vs. 38 (36–40) ms, *p* < 0.0001]. In Cox regression analysis both native T1 and PVS were independently predictive of the primary endpoint and all-cause mortality.

**Conclusion:**

Despite a weak effect of PVS on native T1, its predictive power was not affected in a large, all-comers cohort.

## 1. Introduction

T1 mapping has become a cornerstone of tissue characterization with cardiac magnetic resonance imaging (CMR). The native T1 relaxation time (in ms) differentiates between healthy and diseased myocardium in various clinical scenarios (1, 2). Native T1 is also a predictor of mortality in ischemic as well as nonischemic cardiomyopathies (3–5). The native T1 time reflects fibrotic remodeling of the myocardium and is also influenced by myocardial and interstitial water content (1, 6).

Recent evidence shows that short-term fluctuations in a patient’s volume status impact T1 relaxation times. Luetkens et al. have shown that rapid dehydration and rehydration of healthy individuals significantly change native T1 times (7). The same has been shown for hemodialysis patients who had decreasing T1 times after hemodialysis in two studies (8, 9), although this was contradicted by Graham-Brown et al. who found no such effect (10). As congestion and volume overload are a hallmark of heart failure (HF), a patient’s volume status might confound prognostic information gained from determining native T1 times.

The plasma volume status (PVS), which is derived from anthropometric data and the hematocrit according to Hakim’s formula, is an easily accessible surrogate marker for the patient’s volume status and has been shown to provide prognostic information (11).

It was the aim of the present study to test the hypothesis that the prognostic information obtained from native T1 times is independent of the hydration status as represented by PVS.

## 2. Methods

### 2.1. Study population

The present study population was drawn from a prospective CMR/biobank (BioCVI) all-comers registry of patients who underwent CMR at a single center (Kerckhoff Heart and Thorax Center, Bad Nauheim, Germany) between April 2017 and October 2022. Patients were enrolled in this study if they had a hematocrit measured immediately before the CMR examination.

Clinical indications for CMR included the assessment of myocardial function, ischemia testing, viability testing, and differentiation of cardiomyopathy.

Patients were defined as normal if LVEF was ≥50%, LVEDVi was ≤105 ml/m^2^, and no late gadolinium enhancement (LGE) or perfusion defects were found. Patients were defined as ischemic cardiomyopathy if LVEF was ≤40% and there was ischemic LGE or at least two-vessel disease or ostial LAD disease according to the definition of Felker et al. (12).

Patients were defined as nonischemic cardiomyopathy if LVEF was ≤40%, LVEDVi was ≥105 ml and there was no ischemic LGE, and less than two-vessel disease and no ostial LAD disease or left main disease.

Patients with inflammatory heart disease were excluded to avoid bias by edema.

The registry database contains answers to a standardized questionnaire including symptoms, clinical and familial history, and medication as well as results of a standard CMR protocol with postprocessing and information from a clinical routine follow-up by questionnaire or telephone after 1 year. In addition, a single blood sample for the BioCVI biobank was taken at admission.

All patients gave their written informed consent. The registry was approved by the ethics committee of the University of Giessen.

### 2.2. CMR acquisitions

Standard CMR was performed on a 3 T scanner (Skyra, Siemens Healthineers, Erlangen, Germany) in the head-first supine position with an 18-array coil in agreement with the recommendations of the Society of Cardiovascular Magnetic Resonance (SCMR) (13). The standard protocol included CINE imaging, tissue characterization by T1 and T2 mapping, extracellular volume (ECV) calculation, and LGE as well as—where appropriate—regadenoson perfusion imaging.

### 2.3. SSFP CINE imaging

Retrograde ECG-gated standard steady-state free precision (SSFP) CINE imaging was carried out using the following setting: TE 1.38 ms, TR 3.15 ms, flip angle 50°, bandwidth 962 Hz/px, field of view (FOV) 380 mm, voxel size 1.8 mm × 1.8 mm, slice thickness 8 mm, interslice gap 2 mm, and temporal resolution 30 ms. CINE sequences were generated in 11–15 short-axis views covering the whole ventricle from base to apex and in three long-axis views (two-, three-, and four-chamber views). To compensate for motion artifacts due to breathing or arrhythmias CINE images were acquired using compressed sensing.

### 2.4. Native T1 mapping

Modified look locker sequences [MOLLI 3(2)3(2)5, Goethe CVI approaches®, Frankfurt, Germany]; TE 1.14 ms, TR 3.1 ms, bandwidth 108 Hz/px, FOV 350 mm, voxel size 1.4 mm × 1.4 mm × 8.0 mm, slice thickness 8 mm, adiabatic inversion pulse, 11 inversion times, and ECG-gated antegrade SSFP single-shot readout with 50° flip angle were acquired in three short-axis slices from base to apex following 5 into 3 planning.

### 2.5. T2 mapping

T2 maps were generated before the injection of contrast agent using ECG-gated antegrade T2 prep SSFP sequences generating with the breath-hold technique. Typical parameters were TE 1.34 ms, TR 4.2 ms, flip angle 12°, voxel size 1.8 mm × 1.8 mm, slice thickness 8 mm, and T2 prep with 0, 30, and 55 ms. Three short-axis slices were acquired in the same slice position as with T1 maps from base to apex.

### 2.6. Late gadolinium enhancement

Inversion recovery-segmented gradient echo sequences were acquired 10–15 min after intravenous injection of gadolinium-dota (Dotarem®, Guerbet, Villepinte, France; 0.15 mmol/kg bodyweight) in short-axis and two-, three-, and four-chamber long-axis views. The delay between contrast bolus and acquisition was recorded by the technician. Typical parameters were TE 1.97 ms, TR 3.5 ms, flip angle 20°, bandwidth 289 Hz/px, FOV 370 mm, voxel size 1.3 mm × 1.3 mm × 8.0 mm, and slice thickness 8 mm.

### 2.7. Postprocessing

All analyses were performed on a commercially available workstation (CVI42, Calgary, Canada). For volumetric measurements, automatic contour detection by CVI42 was used excluding trabecularization. Contours were carefully checked by an experienced examiner (AR and JT with level 3 CMR certification from the German Society of Cardiology). End-systolic and end-diastolic volumes were indexed to body surface area (ESVi and EDVi, respectively). Ejection fraction (EF) and longitudinal (GLS), circumferential (GCS), and radial (GRS) strain were calculated as functional parameters.

Global T1 was defined in the midventricular septum outside of LGE regions. A region of interest (ROI) of at least two voxels wide was drawn in an automatically generated parametric T1 map (MyoMaps, Siemens Healthineers, Forchheim, Germany) by an experienced cardiologist (AR and JT), and mean T1 values were calculated. To avoid partial volume effects of the blood pool, the motion correction of the native magnitude pictures was carefully checked and ROI was placed in the center of the septum as recommended by the ConSept method (14).

### 2.8. Calculation of PVS

Plasma volume status was calculated according to the Hakim formula (15) as the degree of deviation from the actual plasma volume (aPV) to the ideal plasma volume (iPV). The aPV was calculated by the hematocrit and the body weight (16):


aPV=[a+(bxbody weight[kg])]x(1−hematocrit)

(*a* = 1,530 in males and 864 in females; *b* = 41 in males and 47.9 in females)

The iPV was calculated using the body weight (16):


iPV=cxbody weight[kg]

(*c* = 39 in males and 40 in females)

Finally, the PVS was calculated by using iPV and aPV (16).


$$PVS=\frac{aPV-iPV}{iPV}\;x\;100\%$$

A PVS > −13% was defined as reference value, a PVS > −4% was found to be associated with poor prognosis in the ValHeFT cohort (17). Both were therefore defined as cut-off points for the present study.

### 2.9. Study endpoints

Study endpoints were defined according to the 2014 ACC/AHA Key Data Elements and Definitions for Cardiovascular Endpoint Events in Clinical Trials (18).

The primary endpoint was defined as a combination of cardiovascular death (CVD) and hospitalization for HF after 1 year.

The secondary endpoint was all-cause mortality after 1 year.

The clinical endpoints were recorded by standardized questionnaires administered either *via* telephone or in writing at least 1 year after the index CMR. All recorded endpoints were adjudicated in an endpoint conference.

### 2.10. Statistics

All metric parameters are presented as median and interquartile range. Categorial variables are presented as absolute frequencies and percentages. Normality of the data was checked by the Shapiro–Wilk test. The Wilcoxon rank-sum test was used to compare groups. Linear regression analysis was performed to examine the correlation between PVS, N-terminal pro-brain natriuretic peptide (NT-pro-BNP), and T1 time. To account for possible heteroscedasticity, we performed robust regression analysis with the Huber-White variance estimator. Univariable Cox proportional hazard analysis was performed to evaluate the association between the study endpoints and CMR or clinical predictors. A multivariable model was fitted for the study endpoint to test the independent prognostic value of the predictors. As a rule of thumb one predictor per 10 events was included. A value of *p* < 0.05 was considered as statistically significant. All tests were computed using Stata 17 (Stata Corp, College Station, Texas, United States).

## 3. Results

Since April 2017, 2,941 patients were included in the registry. As hematocrit values from blood drawn immediately before the examination were only available for 2,183 patients, the remaining 758 patients were excluded. 136 patients were categorized as having active inflammation and were also excluded leaving 2,047 patients for the final analysis.

Clinical and baseline CMR parameters are illustrated in Tables 1, 2. The mean age of the cohort was 63 (52–72) years and 670 patients (33%) were female. One quarter (512 patients, 25%) showed normal findings on CMR, and the remaining 1,535 (75%) had diseased myocardium. About one third of the remaining patients (735 patients, 36%) had ischemic (20%) or non-ischemic cardiomyopathy (16%). Rare cardiomyopathies like hypertrophic cardiomyopathy or non compaction cardiomyopathy were the minority and are summarized as other cardiomyopathies. Borderline findings, which do not fit into the definitions mentioned above were classified as others. The distribution of MR findings and clinical diagnoses is provided in Table 1.

**Table 1:** Baseline characteristics of the entire cohort.

Baseline characteristic	Median [IQR] or *n* (%)
Age, years	63 [52–72]
Female	670 (33)
Body mass index, kg/m^2^	27 [24–30]
Heart rate, beats/min	68 [60–77]
Systolic blood pressure, mmHg	125 [116–139]
Diastolic blood pressure, mmHg	79 [70–82]
Arterial hypertension	1,346 (66)
Chronic kidney disease	308 (15)
Hyperlipidemia	1,024 (50)
Diabetes mellitus	395 (19)
Hematocrit, %	42.8 [39.5–45.7]
NT-pro-BNP, pg./ml	259 [88–1,018]
Troponin, pg./ml	12 [6–27]
eGFR MDRD formula, ml/min/1.73 m^2^	91 [72–110]
CRP, mg/dl	0.2 [0.1–0.6]
MR diagnosis	
Normal findings	512 (25)
Chronic coronary syndrome	468 (23)
Ischemic cardiomyopathy	414 (20.4)
Dilated cardiomyopathy	321 (16)
Other cardiomyopathy	28 (1.4)
Storage disease	12 (0.6)
Pulmonary hypertension/right heart disease	21 (1)
MINOCA	21 (1)
Valvular heart disease	7 (0.3)
Other	168 (8)

CRP, C-reactive protein; eGFR, estimated glomerular filtration rate; IQR, interquartile range; MDRD, modification of diet in renal disease; NT-pro-BNP, N-terminal fragment of pro-brain natriuretic peptide; and MINOCA, myocardial infarction with non-obstructive coronary artery.

**Table 2:** Baseline CMR parameters.

CMR parameter	Median [IQR]
LV-EDVi, ml/m^2^	82 [68–102]
LV-ESVi, ml/m^2^	36 [26–56]
LV-Massi, mg/m^2^	53 [43–65]
RV-EDVi, ml/m^2^	77 [64–91]
LV-EF, %	55 [41–62]
RV-EF, %	51 [43–57]
GLS, %	−16.2 [−19—−12]
GCS, %	−17.5 [−21—−13.0]
T1, ms	1,126 [1,090–1,167]
T2, ms	38 [36–40]
ECV	0.25 [0.23–0.28]

ECV, extracellular volume; EDVi, end diastolic volume index; EF, ejection fraction; ESVi, end systolic volume index; GCS, global circumferential strain; GLS, global longitudinal strain; GRS, global radial strain; IQR, interquartile range; LV, left ventricle; and RV, right ventricle.

### 3.1. PVS and tissue markers

**Figure 1:**
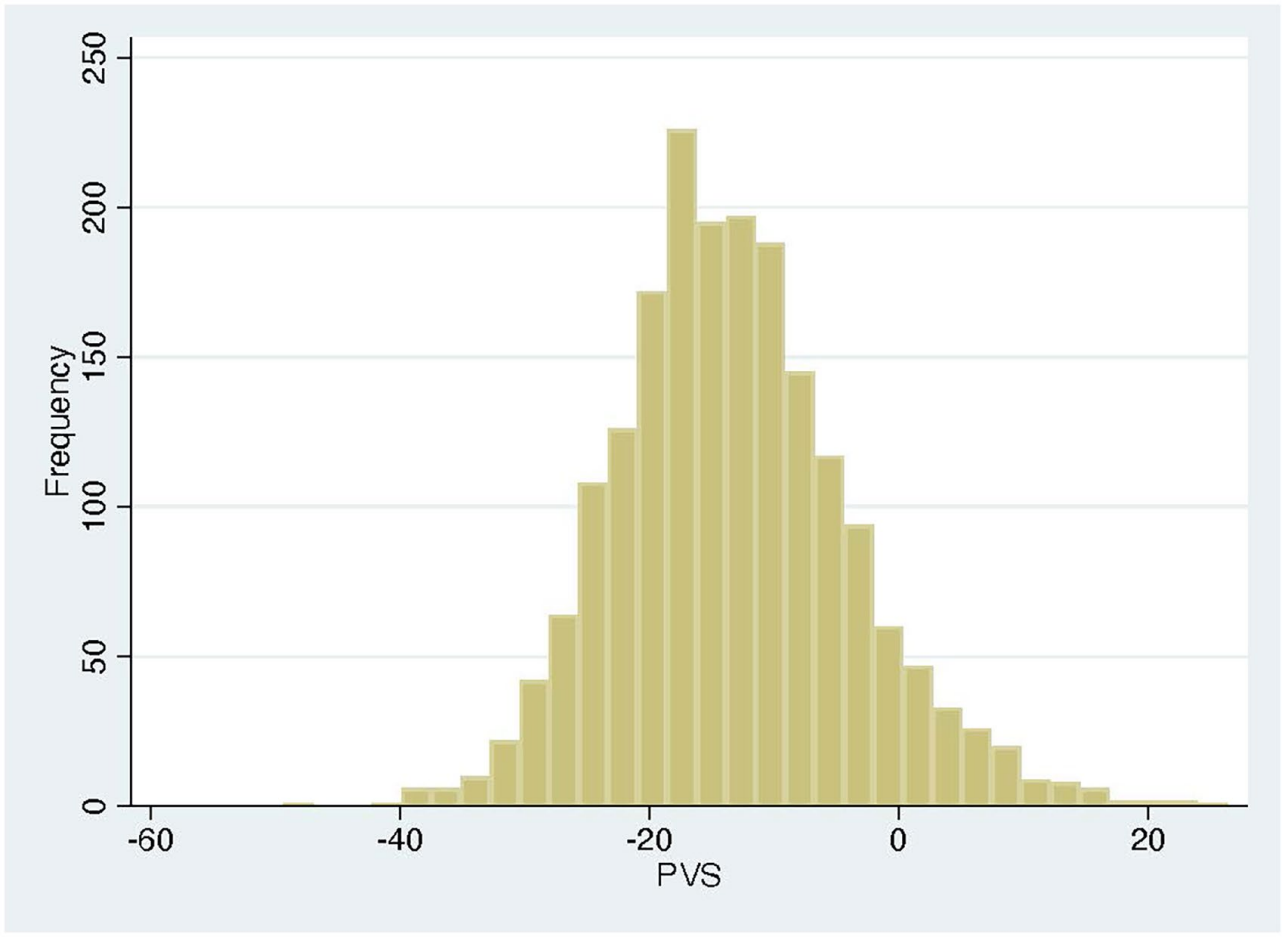
Distribution plot of PVS in the study cohort.

**Figure 2:**
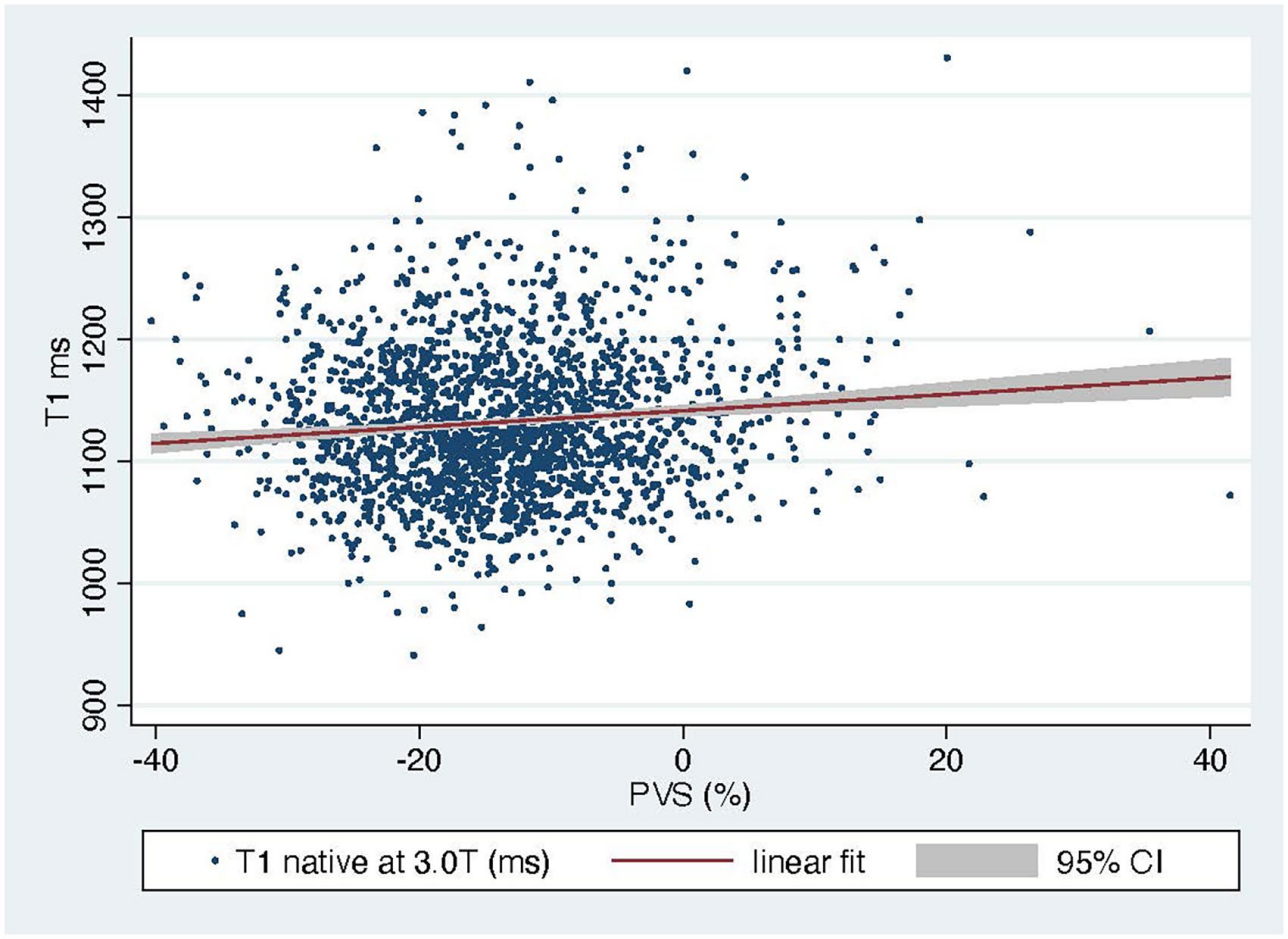
Linear prediction of native T1 by PVS shows a weak but significant relationship.

The PVS distribution of the entire cohort (Figure 1) was very similar to that of the VAL-HeFT cohort. In regression analysis, PVS shows a significant but only weak correlation with native T1 time (*β* = 0.11, *p* < 0.0001) and NT-pro-BNP [*β* = 0.31, *p* < 0.001 and ECV (*β* = 0.23, *p* < 0.0001; compare Figure 2)].

Dichotomizing our cohort at the reference cut-off of −13% yielded 1,110 non-volume-expanded and 937 volume-expanded patients. Native T1, T2, and NT-pro BNP were significantly higher in volume-expanded patients [median (IQR); 1,130 (1,095–1,170) vs. 1,123 (1,086–1,166) ms, *p* < 0.003; 39 (37–40) vs. 38 (36–40) ms, *p* < 0.0001; 318 (111–1,256) vs. 223 (71–794) pg./ml, *p* < 0.0001].

Dichotomizing our cohort at the prognostic cut off of −4% yielded 297 volume overloaded patients; here, the differences of native T1, T2 and NT-pro-BNP were as follows [1,138 (1,100–1,183) vs. 1,124 (1,087–1,165) ms; *p* < 0.0001; 39 (37–41) vs. 38 (36–40) ms, *p* < 0.0007; 712 (156–2,256) vs. 237 (80–795) pg./ml; *p* < 0.0001]. Further clinical and CMR parameters in volume-overloaded and-contracted patients are shown in Table 3.

**Table 3:** Comparison of CMR parameters in patients with pathological PVS and normal PVS in the entire cohort.

Parameter	PVS > −4% median [IQR]	PVS ≤ − 4% median [IQR]	*p*
LV-EF, %	54 [40–62]	55 [41–62]	<0.93
GLS, %	−16.3 [−19.2—−11.6]	−16.3 [−19.3—−12.2]	<0.772
RV-EF, %	51 [43–58]	51 [43–57]	<0.512
LV-EDVi, ml/m^2^	82 [69–100]	82 [67–103]	<0.87
RV-EDVi, ml/m^2^	78 [64–90]	76 [64–91]	<0.89
Native T1 time, ms	1,138 [1,100–1,183]	1,124 [1,087–1,165]	<0.0001
ECV	0.27 [0.25–0.30]	0.25 [0.22–0.27]	<0.0001
T2 time, ms	39 [37–41]	38 [36–40]	<0.0007
Nt-pro-BNP, pg./ml	712 [156–2,257]	237 [80–795]	<0.0001
Parameter	PVS > −13% median [IQR]	PVS ≤ −13% median [IQR]	*p*
LV-EF, %	56 [45–63]	54 [38–62]	<0.0002
GLS, %	−16.7 [−19.6—−12.8]	−15.7 [−18.8—−11.5]	<0.0001
RV-EF, %	52 [45–58]	50 [43–56]	<0.0001
LV-EDVi, ml/m^2^	79 [67–98]	84 [69–106]	<0.0002
RV-EDVi, ml/m^2^	76 [62–89]	77 [65–93]	<0.01
Native T1 time, ms	1,130 [1,095–1,170]	1,123 [1,086–1,166]	<0.003
ECV	0.26 [0.24–0.29]	0.23 [0.22–0.26]	<0.0001
T2 time, ms	39 [37–40]	38 [36–40]	<0.0001
Nt-pro-BNP, pg./ml	318[111–1,256]	223 [71–794]	<0.0001

ECV, extracellular volume; EDVi, end diastolic volume index; EF, ejection fraction; ESVi, end systolic volume index; GLS, global longitudinal strain; IQR, interquartile range; LV, left ventricle; RV, right ventricle; SD, standard deviation, and PVS, plasma volume status.

Three-hundred and twelve patients had an eGFR less than 60 and were included in a subanalysis of patients with renal failure. Of these patients, 99 had elevated PVS above the prognostic cut off of −4%. Native T1 was higher in these patients compared to those with PVS less than −4% but the difference did not reach statistical significance (1,161 ± 71 ms vs. 1,148 ± 69 ms, *p* = 0.12).

In regression analysis, PVS shows a significant but only weak correlation with native T1 time (*β* = 0.11, *p* < 0.0001) and NT-pro-BNP (*β* = 0.31, *p* < 0.001; compare Figure 2).

### 3.2. Outcome analysis

One-year follow-up was completed in 1,363 (66%) cases after a mean of 430 days. T he primary endpoint of CV death and hospitalization for heart failure occurred in 46 patients (3%).

The secondary endpoint of all-cause mortality was noted in 19 (0.6%) cases.

In a univariable Cox regression model for the primary endpoint as well as the secondary endpoint both PVS and native T1 showed a significant effect on the endpoints.

**Figure 3:**
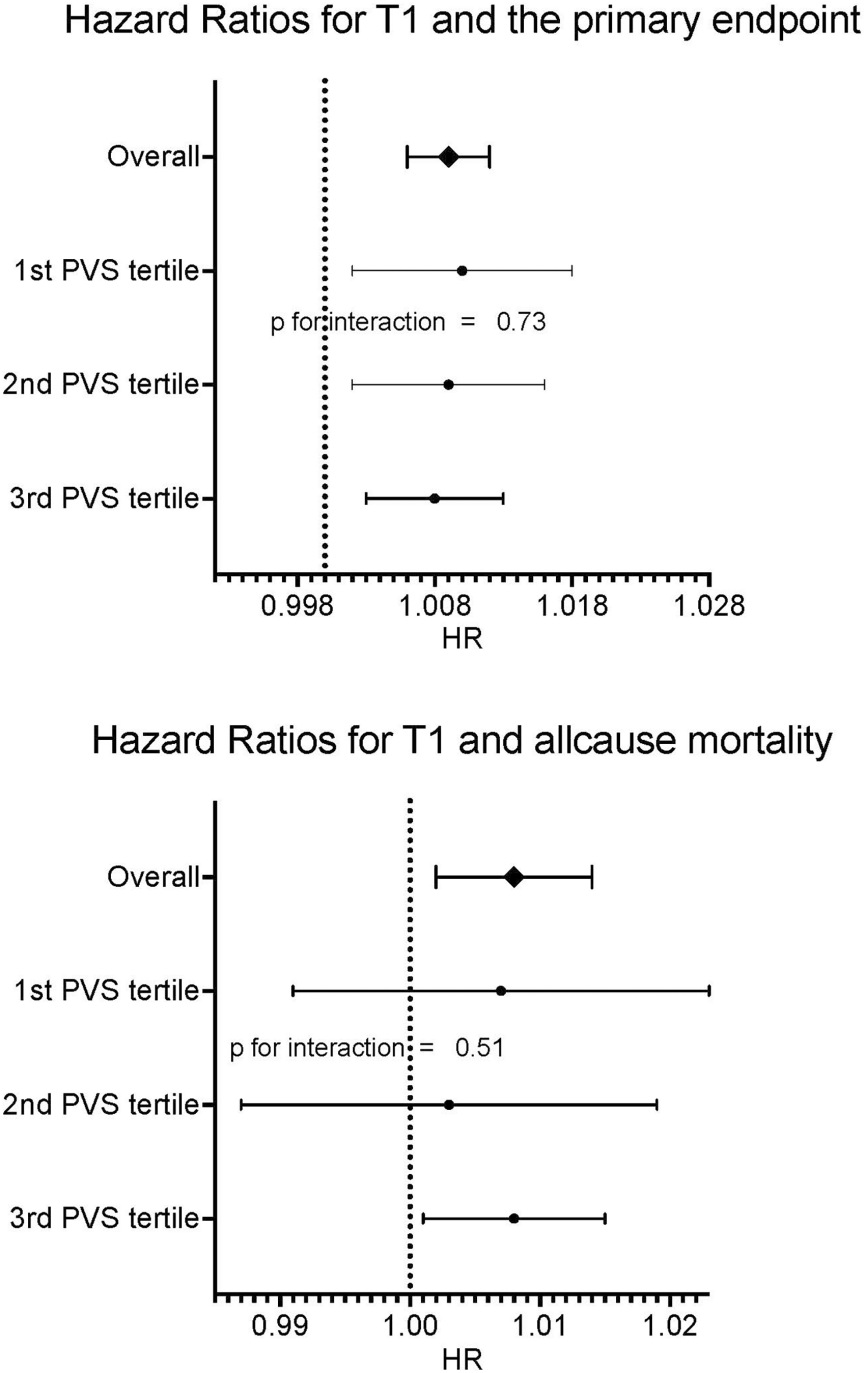
Forrest plots for primary and secondary endpoints according to PVS tertiles. No relevant interaction between PVS tertiles and native T1 could be found.

In a multivariable model for the primary endpoint cardiovascular death/heart failure hospitalizations, which also included EF, ECV, ESVi, GLS, and age, both native T1 time and PVS were independent predictors, with the interaction term being not significant (Table 4, compare Figure 3).

**Table 4:** Cox regression analysis for the primary endpoint of cardiovascular death/heart failure hospitalizations.

Variable	HR	95% CI		*p*
		Lower	Upper	
Univariable analysis				
T1 time	1.009	1.006	1.013	0.0001
PVS	1.069	1.039	1.099	0.0001
EF	0.957	0.941	0.973	0.0001
ESVi	1.015	1.010	1.020	0.0001
Age	1.029	1.005	1.053	0.017
GLS	1.15	1.085	1.222	0.0001
ECV	8.93	1.484	53.724	0.017
Multivariable analysis				
T1 time	1.005	1.00054	1.009	0.028
PVS	1.054	1.026	1.083	0.0001
EF	0.975	0.947	1.003	0.083
ESVi	1.005	0.996	1.015	0.282
Age	1.016	0.992	1.041	0.189
Multivariable analysis GLS for EF				
T1-time	1.005	1.00054	1.01	0.029
PVS	1.053	1.025	1.083	0.0001
GLS	1.059	0.974	1.152	0.179
ESVi	1.0008	1.001	1.016	0.037
Age	1.012	0.986	1.04	0.354
Multivariable analysis GLS for EF, ECV for ESVi				
T1 time	1.005	0.999	1.01	0.083
PVS	1.054	1.024	1.083	0.001
GLS	1.108	1.037	1.184	0.002
ECV	1.55	0.045	52.20	0.808
Age	1.005	0.980	1.031	0.691

CI, confidence interval; ECV, extracellular volume, GLS, global longitudinal strain; PVS, plasma volume status; HR, hazard ratio; EF, ejection fraction; and ESVi, endsystolic volume indexed for body surface area.

**Figure 4:**
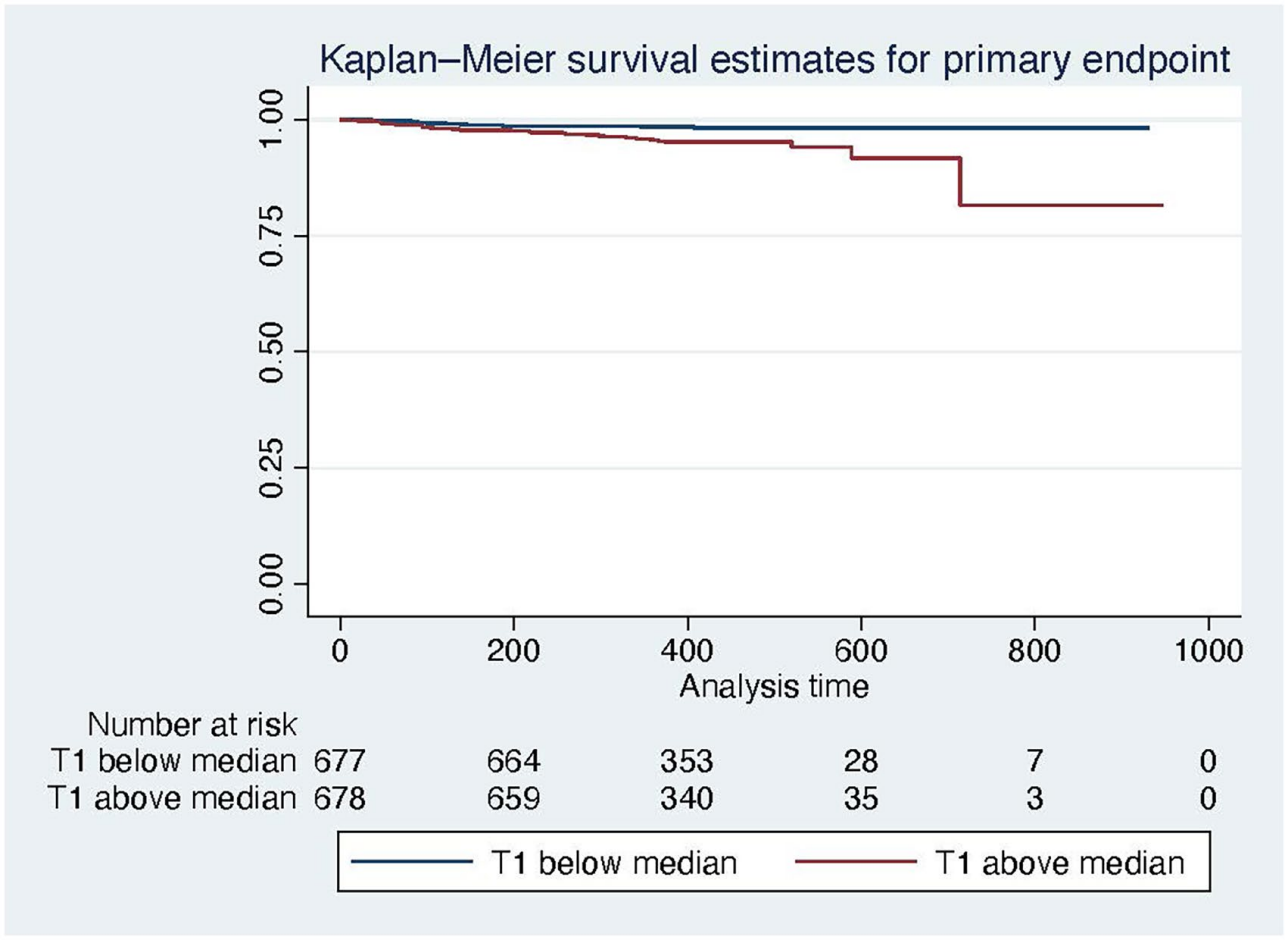
Kaplan Meier survival curves for the primary endpoint for patients below (blue) and above (red) the median native T1 time.

In a multivariable model with all-cause mortality as outcome variable and native T1 and PVS as independent variables, both were independently predictive of mortality, with the interaction term being not significant (Table 5, compare Figure 3). Kaplan Meier curves show, that patients with native T1 above the median have significantly shorter event free survival with respect to the primary endpoint (compare Figure 4).

**Table 5:** Cox regression analysis for the secondary endpoint all-cause mortality.

Variable	HR	95% CI		*p*
		Lower	Upper	
Univariable analysis				
Native T1 time	1.008	1.002	1.014	0.007
PVS	1.089	1.044	1.136	0.0001
Multivariable analysis				
Native T1 time	1.006	1.001	1.013	0.042
PVS	1.076	1.033	1,121	0.0001

CI, confidence interval; PVS, plasma volume status; and HR, hazard ratio.

## 4. Discussion

Native T1 time is an essential parameter for the diagnosis of various cardiac diseases and can be used to estimate prognosis in ischemic as well as non-ischemic heart disease (1, 4, 19). Recent reports have shown that short-term fluctuations of a patient’s volume status by hydration or hemodialysis can influence native T1. The volume status as confounder of native T1 might challenge its diagnostic and prognostic value. The aim of our study was to show that native T1 is predictive of MACE (CVD and hospitalization for HF after 1 year) and mortality irrespective of volume status measured by PVS. The main findings are:

1. There is a significant but only weak linear relation between PVS as a surrogate marker of volume status and native T1 time.
2. Volume-expanded patients with a PVS > −13% have significantly elevated tissue markers compared to volume-contracted patients; however, this difference is small.
3. Despite this marginal influence of volume status on the native T1 time, it remains independently predictive of MACE and all-cause mortality.

Native T1 maps are widely used and are a robust and reproducible method to estimate T1 relaxation time of the myocardium (20, 21). The T1 relaxation time is most often increased by the collagen and/or water content of the myocardium (22, 23) and less frequently by amyloid deposits. Interstitial or intracellular accumulation of lipids and iron will decrease native T1 (5, 24).

While the influence of free water on the native T1 is usually a short-term effect, it is fibrosis that has a long-lasting influence on T1 times and determines prognosis (4, 19). With respect to the prognostic information of native T1, the influence of short-term fluctuations of water on the volume status must be considered a confounding factor.

Plasma volume status, determined according to Hakim’s formula, is a surrogate marker of volume status that can easily be derived from anthropometric data and hematocrit (11, 15). PVS has been validated with directly measured volume status and found to be predictive of prognosis in a substudy of the Val-HeFT cohort (17). Two cut-off points were derived in this study: −13%, which has been found to be the ideal PVS and hence the dividing line between volume expansion and contraction, and −4%, above which patients are volume overloaded and prognosis in heart failure deteriorates. The distribution of volume status in our cohort was similar to that in the VAL-HeFT study (Figure 1), and the relationship of PVS and NT-pro-BNP is also in very good agreement with that published in the Val-HeFT cohort with a *β* coefficient of 0.19 (*p* = 0.0001).

Applying both cut-offs to our cohort we find indeed higher native T1 in volume-loaded patients. Patients above the reference value of −13% have 13 ms higher T1 times than patients below. This effect size is in good agreement with that of Luetkens et al., who found an 18 ms difference (at 1.5 T) in healthy volunteers after dehydration and rehydration (7). It is also similar to data from Rankin et al. at 3 T, who found a difference in native T1 of 21 ms after hemodialysis (9). Further, our data are in good agreement with data from Lurz et al., who found a similar confounding of T1 and ECV by increased tissue water as a consequence of inflammation (25). In this respect, our data confirm in a large cohort of patients that the volume status has indeed a small influence on native T1. Also T2 times in this cohort were also higher in volume expanded patients, which further supports our hypothesis.

Even in the absence of overt heart failure, MI, or non-ischemic cardiomyopathy, cardiovascular risk factors can enhance interstitial fibrosis and hence increase native T1 (26). This translates into reduced myocardial function even in visually normal EF (27, 28). With increasing fibrosis it also affects prognosis (4, 19).

Hyperhydration is a distinctive feature of worsening chronic HF. The volume overload induces dyspnea and peripheral edema and leads to hospitalization more frequently than does low cardiac output (29, 30). Congestion might be clinically silent, and peripheral edema or pulmonary vein congestion are not obligatory (31). From this standpoint, the confounding effect of excessive water might be especially pronounced in the group of cardiomyopathy patients in which, conversely, the prognostic information of T1 is even more important.

However, the clinical implications of T1 are not critically affected by the volume T1 relationship. R2 in regression analysis was only 0.02, meaning that only 2% of the variance of native T1 values in our cohort can be attributed to volume fluctuations.

Most importantly, the minimal effect of volume status on T1 times did not affect the prognostic implication of native T1. In univariate analysis both native T1 and PVS were predictive of both MACE and all-cause mortality. In multivariable analysis, T1 and PVS were independently predictive of MACE. Both parameters were independently predictive of death. The interaction term between native T1 and PVS was not significant with respect to both MACE and all-cause mortality.

Myocardial fibrosis causes disintegration of the highly organized myocardial interstitium, resulting in impaired transduction of force and increased myocardial stiffness that together mediate systolic and diastolic dysfunction (26, 27, 32). Myocardial fibrosis is also a trigger of ventricular arryhthmia (27, 32). Consequently, both histological and imaging studies of fibrosis have shown that increased fibrosis is associated with poor clinical outcome (4, 19, 33, 34). In principle, CMR offers three tools to detect fibrosis: LGE, native T1, and ECV. While LGE reflects focal fibrosis, especially replacement fibrosis, T1 and ECV account for diffuse fibrosis (1, 32). While ECV quantifies the extracellular space, native T1 is also influenced by intracellular accumulation of water. In our study, ECV was also predictive on the primary endpoint in univariable analysis. Replacing ESVi by ECV in the original multivariable analysis, ECV is not independently predictive of the primary endpoint while there is still a trend for native T1. Nevertheless, native T1 was shown to correlate well with collagen volume fraction on endomyocardial biopsy samples providing high reproducibility (22). Also, native T1 is easily assessable even in high-throughput screening protocols without contrast agent.

Our study confirms the prognostic value of T1 in a large, all-comers cohort of patients with a comparatively high proportion of low-risk patients and normal findings on MR, which underlines the prognostic importance even outside specially selected cohorts of cardiomyopathy patients. Further, we demonstrated that the prognostic information conferred by T1 is not confounded by volume fluctuations.

### 4.1. Limitations

Our study has some limitations. The first limitation is the low number of endpoints reached during the observation period, which reduces the power of our findings. Only 19 patients died during follow up. This is due to the low-risk character of our cohort. Further, PVS is only a surrogate marker of volume expansion or contraction, although it has been well validated against direct volume quantification and its prognostic value has been shown (17). The advantage of this approach is its easy application in the typical, routine clinical setting of a large, all-comers cohort; therefore, we think it justified to generalize our findings to the general population.

### 4.2. Conclusion

Our data confirm the prognostic value of native T1 for MACE and mortality. Despite a weak effect of volume fluctuation on native T1, this does not hamper its prognostic impact.

## Data availability statement

The raw data supporting the conclusions of this article will be made available by the authors, without undue reservation.

## Ethics statement

The studies involving human participants were reviewed and approved by ethics committee of the Justus Liebig University, Gießen, Germany. The patients/participants provided their written informed consent to participate in this study.

## Author contributions

AR, JT, UF-R, and JW conceived and designed the study. CaH, JT, SK, and JW acquired clinical data. CaH, JT, JW, and AR acquired and analyzed imaging data. AR and UF-R executed the statistical analysis. JT drafted the manuscript. AR, EN, VP, CaH, UF-R, SK, ChH, and JT revised and amended critical parts of the manuscript. All authors contributed to the interpretation of the data and approved the final version of this manuscript.

## Conflict of interest

The authors declare that the research was conducted in the absence of any commercial or financial relationships that could be construed as a potential conflict of interest.

## Publisher’s note

All claims expressed in this article are solely those of the authors and do not necessarily represent those of their affiliated organizations, or those of the publisher, the editors and the reviewers. Any product that may be evaluated in this article, or claim that may be made by its manufacturer, is not guaranteed or endorsed by the publisher.

## References

[1] Haaf P Garg P Messroghli DR Broadbent DA Greenwood JP Plein S. Cardiac T1 mapping and extracellular volume (ECV) in clinical practice: a comprehensive review. J Cardiovasc Magn Reson. (2017) 18:89. doi: 10.1186/s12968-016-0308-4, PMID: 27899132 PMC5129251

[2] Taylor AJ Salerno M Dharmakumar R Jerosch-Herold M. T1 mapping: basic techniques and clinical applications. JACC Cardiovasc Imaging. (2016) 9:67–81. doi: 10.1016/j.jcmg.2015.11.005, PMID: 26762877

[3] Puntmann VO Carerj ML Wieters I Fahim M Arendt C Hoffmann J. Outcomes of cardiovascular magnetic resonance imaging in patients recently recovered from coronavirus disease 2019 (COVID-19). JAMA Cardiol. (2020) 5:1265–73. doi: 10.1001/jamacardio.2020.3557, PMID: 32730619 PMC7385689

[4] Puntmann VO Carr-White G Jabbour A Yu CY Gebker R Kelle S. T1-mapping and outcome in nonischemic cardiomyopathy: all-cause mortality and heart failure. JACC Cardiovasc Imaging. (2016) 9:40–50. doi: 10.1016/j.jcmg.2015.12.001, PMID: 26762873

[5] Patel AR Kramer CM. Role of cardiac magnetic resonance in the diagnosis and prognosis of nonischemic cardiomyopathy. JACC Cardiovasc Imaging. (2017) 10:1180–93. doi: 10.1016/j.jcmg.2017.08.005, PMID: 28982571 PMC5708889

[6] Kammerlander AA Marzluf BA Zotter-Tufaro C Aschauer S Duca F Bachmann A. T1 mapping by CMR imaging: from histological validation to clinical implication. JACC Cardiovasc Imaging. (2016) 9:14–23. doi: 10.1016/J.JCMG.2015.11.002, PMID: 26684970

[7] Luetkens JA Voigt M Faron A Isaak A Mesropyan N Dabir D. Influence of hydration status on cardiovascular magnetic resonance myocardial T1 and T2 relaxation time assessment: an intraindividual study in healthy subjects. J Cardiovasc Magn Reson. (2020) 22:63. doi: 10.1186/s12968-020-00661-9, PMID: 32892751 PMC7487526

[8] Kotecha T Martinez-Naharro A Yoowannakul S Lambe T Rezk T Knight DS. Acute changes in cardiac structural and tissue characterisation parameters following haemodialysis measured using cardiovascular magnetic resonance. Sci Rep. (2019) 9:1388. doi: 10.1038/s41598-018-37845-4, PMID: 30718606 PMC6362126

[9] Rankin AJ Mangion K Lees JS Rutherford E Gillis KA Edy E. Myocardial changes on 3T cardiovascular magnetic resonance imaging in response to haemodialysis with fluid removal. J Cardiovasc Magn Reson. (2021) 23:125. doi: 10.1186/s12968-021-00822-4, PMID: 34758850 PMC8580743

[10] Graham-Brown MPM Rutherford E Levelt E March DS Churchward DR Stensel DJ. Native T1 mapping: inter-study, inter-observer and inter-center reproducibility in hemodialysis patients. J Cardiovasc Magn Reson. (2017) 19:21. doi: 10.1186/s12968-017-0337-7, PMID: 28238284 PMC5327541

[11] Grodin JL Philips S Mullens W Nijst P Martens P Fang JC. Prognostic implications of plasma volume status estimates in heart failure with preserved ejection fraction: insights from TOPCAT. Eur J Heart Fail. (2019) 21:634–42. doi: 10.1002/ejhf.1407, PMID: 30714658

[12] Felker GM Shaw LK O’Connor CM. A standardized definition of ischemic cardiomyopathy for use in clinical research. J Am Coll Cardiol. (2002) 39:210–8. doi: 10.1016/s0735-1097(01)01738-7, PMID: 11788209

[13] Kramer CM Barkhausen J Bucciarelli-Ducci C Flamm SD Kim RJ Nagel E. Standardized cardiovascular magnetic resonance imaging (CMR) protocols: 2020 update. J Cardiovasc Magn Reson. (2020) 22:17. doi: 10.1186/s12968-020-00607-1, PMID: 32089132 PMC7038611

[14] Rogers T Dabir D Mahmoud I Voigt T Schaeffter T Nagel E. Standardization of T1 measurements with MOLLI in differentiation between health and disease--the ConSept study. J Cardiovasc Magn Reson. (2013) 15:78. doi: 10.1186/1532-429X-15-78, PMID: 24025486 PMC3847466

[15] Ismail N Neyra R Hakim R. “Plasmapheresis,” in Handbook of Dialysis. *3*. eds. Daugirdas J. Blake PG. Ing TS. (Philadelphia: Lippincott, Williams and Wilkins), (2007) 231–62.

[16] Fudim M Miller WL. Calculated estimates of plasma volume in patients with chronic heart failure—comparison to measured volumes. J Card Fail. (2018) 24:553–60. doi: 10.1016/J.CARDFAIL.2018.07.462, PMID: 30098381 PMC6196104

[17] Ling HZ Flint J Damgaard M Bonfils PK Cheng AS Aggarwal S. Calculated plasma volume status and prognosis in chronic heart failure. Eur J Heart Fail. (2015) 17:35–43. doi: 10.1002/ejhf.193, PMID: 25469484

[18] Hicks KA Tcheng JE Bozkurt B Chaitman BR Cutlip DE Farb A. 2014 ACC/AHA key data elements and definitions for cardiovascular endpoint events in clinical trials. Circulation. (2015) 132:302–61. doi: 10.1161/CIR.0000000000000156, PMID: 25547519

[19] Puntmann VO Carr-White G Jabbour A Yu CY Gebker R Kelle S. Native T1 and ECV of noninfarcted myocardium and outcome in patients with coronary artery disease. J Am Coll Cardiol. (2018) 71:766–78. doi: 10.1016/j.jacc.2017.12.020, PMID: 29447739

[20] Kellman P Hansen MS. T1-mapping in the heart: accuracy and precision. J Cardiovasc Magn Reson. (2014) 16:2–22. 24387626 10.1186/1532-429X-16-2 PMC3927683

[21] Roujol S Weingärtner S Foppa M Chow K Kawaji K Ngo LH. Accuracy, precision, and reproducibility of four T1 mapping sequences: a head-to-head comparison of MOLLI, ShMOLLI, SASHA, and SAPPHIRE. Radiology. (2014) 272:683–9. doi: 10.1148/radiol.14140296, PMID: 24702727 PMC4263641

[22] Nakamori S Dohi K Ishida M Goto Y Imanaka-Yoshida K Omori T. Native T1 mapping and extracellular volume mapping for the assessment of diffuse myocardial fibrosis in dilated cardiomyopathy. JACC Cardiovasc Imaging. (2018) 11:48–59. doi: 10.1016/j.jcmg.2017.04.006, PMID: 28624408

[23] de Meester de Ravenstein C Bouzin C Lazam S Boulif J Amzulescu M Melchior J. Histological validation of measurement of diffuse interstitial myocardial fibrosis by myocardial extravascular volume fraction from modified look-locker imaging (MOLLI) T1 mapping at 3 T. J Cardiovasc Magn Reson. (2015) 17:48. doi: 10.1186/s12968-015-0150-0, PMID: 26062931 PMC4464705

[24] Kramer CM. Role of cardiac MR imaging in cardiomyopathies. J Nucl Med. (2015) 56:39S–45S. doi: 10.2967/jnumed.114.142729, PMID: 26033902 PMC4465292

[25] Lurz JA Luecke C Lang D Besler C Rommel KP Klingel K. CMR-derived extracellular volume fraction as a marker for myocardial fibrosis: the importance of coexisting myocardial inflammation. JACC Cardiovasc Imaging. (2018) 11:38–45. doi: 10.1016/j.jcmg.2017.01.025, PMID: 28412435

[26] Pezel T Viallon M Croisille P Sebbag L Bochaton T Garot J. Imaging interstitial fibrosis, left ventricular remodeling, and function in stage a and B heart failure. JACC Cardiovasc Imaging. (2021) 14:1038–52. doi: 10.1016/j.jcmg.2020.05.036, PMID: 32828781

[27] Schelbert EB Butler J Diez J. Why clinicians should care about the cardiac Interstitium. JACC Cardiovasc Imaging. (2019) 12:2305–18. doi: 10.1016/j.jcmg.2019.04.025, PMID: 31422140

[28] Korosoglou G Giusca S Montenbruck M Patel AR Lapinskas T Götze C. Fast strain-encoded cardiac magnetic resonance for diagnostic classification and risk stratification of heart failure patients. JACC Cardiovasc Imaging. (2021) 14:1177–88. doi: 10.1016/j.jcmg.2020.10.024, PMID: 33454266

[29] McDonagh TA Metra M Adamo M Gardner RS Baumbach A Böhm M. 2021 ESC guidelines for the diagnosis and treatment of acute and chronic heart failure: Developed by the Task Force for the diagnosis and treatment of acute and chronic heart failure of the European Society of Cardiology (ESC) with the special contribution of the Heart Failure Association (HFA) of the ESC. Eur Heart J. (2021) 42:3599–726. doi: 10.1093/eurheartj/ehab368, PMID: 34447992

[30] Dupont M Mullens W Tang WHW. Impact of systemic venous congestion in heart failure. Curr Heart Fail Rep. (2011) 8:233–41. doi: 10.1007/s11897-011-0071-7, PMID: 21861070

[31] Stevenson LW Perloff JK. The limited reliability of physical signs for estimating hemodynamics in chronic heart failure. Continuing Edu Opportunities Physicians Period. (1989) 261:884–8. doi: 10.1001/jama.1989.03420060100040, PMID: 2913385

[32] Halliday BP Prasad SK. The Interstitium in the hypertrophied heart. JACC Cardiovasc Imaging. (2019) 12:2357–68. doi: 10.1016/j.jcmg.2019.05.033, PMID: 31542527

[33] Azevedo CF Nigri M Higuchi ML Pomerantzeff PM Spina GS Sampaio RO. Prognostic significance of myocardial fibrosis quantification by histopathology and magnetic resonance imaging in patients with severe aortic valve disease. J Am Coll Cardiol. (2010) 56:278–87. doi: 10.1016/j.jacc.2009.12.074, PMID: 20633819

[34] Flett AS Hayward MP Ashworth MT Hansen MS Taylor AM Elliott PM. Equilibrium contrast cardiovascular magnetic resonance for the measurement of diffuse myocardial fibrosis: preliminary validation in humans. Circulation. (2010) 122:138–44. doi: 10.1161/CIRCULATIONAHA.109.930636, PMID: 20585010

